# Diagnosis and treatment of liver metastases of parathyroid carcinoma

**DOI:** 10.3389/fendo.2022.982972

**Published:** 2022-10-11

**Authors:** Chaoyun Su, Junwei Zhang, Huayu Yang, Yiyao Xu, Xin Lu

**Affiliations:** ^1^ Department of General Surgery, Beijing Aerospace General Hospital, Beijing, China; ^2^ Department of Liver Surgery, State Key Laboratory of Complex Severe and Rare Diseases, Peking Union Medical College Hospital, Chinese Academy of Medical Science and Peking Union Medical College, Beijing, China

**Keywords:** parathyroid carcinoma, liver metastases, diagnosis, treatment, case report

## Abstract

**Introduction:**

Parathyroid carcinoma (PC) is a very rare endocrine malignancy occurring in less than 1% of all cases of primary hyperparathyroidism (pHPT). The liver is the second most common target organ for distant metastases of PC, but no guidelines are available for the diagnosis and treatment of liver metastases. In this study, we attempted to summarize the characteristics of the diagnosis and treatment of liver metastases based on our patients and other cases reported in the literature.

**Materials and methods:**

The files of all patients diagnosed with PC with liver metastases summarized at our center between 2000 and 2022 were reviewed, and three datasets from Medline, Web of Science, and Embase were systematically searched to identify relevant articles.

**Results:**

Three patients with liver metastases from our center and 11 patients from the literature were included in the study. All patients had pHPT with borderline remission of hypercalcemia after each operation. A total of 71.4% of the patients’ liver lesions were found by abdominal CT scans, while 35.7% were found by MRI, PET-CT, and fine-needle aspiration biopsy (FNAB), which were also helpful for diagnosis. Eight of nine patients (88.9%) who underwent surgery, radiofrequency ablation (RFA), or transcatheter arterial embolization (TAE) were alive, and only one postoperative patient died after a follow-up of 60 months.

**Conclusions:**

PC is a rare malignant tumor prone to recurrence and metastasis, and postoperative reviews should be carried out routinely. Abnormally elevated parathyroid hormone (PTH) and serum calcium can indicate recurrence or metastasis. Enhanced CT and MRI can provide valuable support for the diagnosis of liver metastases, but whether [18F]FDG-PET-CT, [18F]FCH-PET-CT, or [11C]choline-PET-CT can be used as a diagnostic basis requires further study. Resection of liver metastases, segmental hepatectomy, or RFA can significantly improve patients’ symptoms.

## Introduction

Parathyroid carcinoma (PC) is a very rare endocrine tumor cause of primary hyperparathyroidism (pHPT), with an annual incidence of less than 1% of all cases of hyperparathyroidism and for only 0.005% of all cancers (1, 2). Most (90%) PC cases are hormonally functional, but the clinical manifestations of PC are diverse, which complicates its diagnosis. Whether initial surgery can completely remove the tumor is the key to the prognosis. Improving the preoperative diagnosis rate and surgeons’ awareness is meaningful to establish a better method to treat PC.

The high rate of recurrence and distant metastases are another challenge of PC, and the most common distant metastases are in the lungs and liver (3, 4). To date, no standard method has been established for the diagnosis and treatment of liver metastases. Only a few case reports on liver metastases of PC are available, and treatment is very difficult. We summarized the symptoms, imaging, and treatment of PC patients with liver metastases in our center and reviewed the literature related to PC. In this study, we attempted to summarize the diagnostic points and treatment options for liver metastases based on our patients and other cases reported in the literature.

## Materials and methods

In this retrospective study, we reviewed the archive files of all patients diagnosed with PC with liver metastases at the Peking Union Medical College Hospital (PUMCH) between 2000 and 2022. Primary hyperparathyroidism was confirmed biochemically by elevated serum calcium and parathyroid hormone with/without concomitant symptoms (bone pain, nephrolithiasis, pathological fractures, polydipsia and polyuria, digestive symptoms, etc.). PC and liver metastases were diagnosed by professional pathologists based on pathological examination of resected specimens. Some of the diagnostic features are listed below: (1) sheets or lobules of tumor cells with interspersed fibrous bands; (2) mitotic figures; (3) necrosis; (4) capsular invasion; and (5) vascular invasion (5). The largest single diameter from the pathology report was utilized as the tumor size. The age of onset was recorded according to the first diagnosis of liver metastases of PC. Recurrence was defined as hypercalcemia presenting after a normal serum calcium period at more than 6 months after the surgery (6). Normal reference ranges for indices: serum calcium: 2.13–2.70 mmol/L, PTH: 15–65 pg/ml.

### Systematic review

We developed the following search strategy to identify relevant studies of PC with liver metastases. Three datasets from Medline, Web of Science and Embase were retrieved. The search was limited to humans, and the language was restricted to English. All types of publications were included. The following retrieval strategy was used: [(Neoplasm, Parathyroid) OR (Parathyroid Neoplasm) OR (Neoplasms, Parathyroid) OR (Parathyroid Adenoma) OR (Adenoma, Parathyroid) OR (Adenomas, Parathyroid) OR (Parathyroid Adenomas) OR (Parathyroid Carcinoma) OR (Carcinoma, Parathyroid) OR (Carcinomas, Parathyroid) OR (Parathyroid Carcinomas) OR (Cancer of Parathyroid) OR (Parathyroid Cancers) OR (Cancer of the Parathyroid) OR (Parathyroid Cancer) OR (Cancer, Parathyroid) OR (Cancers, Parathyroid)] and [(liver metastasis) OR (liver metastases) OR (liver metastasis tumor) OR (hepatic metastases) OR (hepatic metastasis) OR (metastatic liver) OR (metastatic liver tumor)]. The references of the included studies were also screened to retrieve related papers. The last search was performed on 8 August 2022. Two investigators (CS and JZ) completed the search independently.

### Study selection and inclusion criteria

Two authors (CS and JZ) independently used the following standardized process to screen the papers, review their full texts, and select the articles meeting the inclusion criteria. Studies were identified as follows: (1) duplicate studies were excluded; (2) titles were screened, and only articles that were relevant to PC were selected; and (3) abstracts and full texts were reviewed if studies could not be excluded by titles alone. The inclusion criteria were as follows: (1) studies investigating PC and liver metastasis; (2) human studies; (3) studies published in English; and (4) studies with available relevant data. The exclusion criteria were as follows: (1) animal studies; (2) non-English studies; and (3) studies with overlapping data.

### Data extraction

In view of the rarity of PC with liver metastases, we summarized the clinical data of the cases reported in the included studies, including age, sex, time from initial diagnosis to liver metastases, liver mass location and size, detection modality, serum calcium and PTH levels before and after treatment, treatment methods, and prognosis. Data from retrospective studies on liver metastases were also collected. Combined with our cases, we tried to provide methods for the diagnosis and treatment of PC with liver metastases.

## Results

### Cases at our hospital

We searched all medical records in our hospital from 1 January 2000 to 30 June 2022, and retrieved a total of 254 PC patients. Among them, 3 (1.18%) had developed liver metastases, and all three were treated surgically in our hospital with 3–96 months of follow-up alive. Their clinical presentations, including maximum serum calcium and PTH levels, recurrence times, and treatment methods for distant metastases, are listed in **Table 1**. The specific diagnoses and treatments are organized as follows.

**Table 1:** Clinical presentation of the three patients.

	Patient 1	Patient 2	Patient 3
Age (years)	43	50	37
Gender	Male	Male	Female
Presentation	Polydipsia, polyuria, renal stone, and rib pain	Knee pain, polydipsia, and polyuria	Osteoporosis, femur fracture, polydipsia, and polyuria
Height loss (mm)	50	30	40
Maximum Ca (mmol/L)	3.74	4.40	3.99
Maximum PTH (pg/ml)	1,300	2,004	2,076
PTH above normal level	20 times	31 times	30 times
Size of PC (mm)	50	23	45
Lymph node metastasis	No	Yes	Yes
Recurrent times	2	7	3
Time for liver metastasis (months)	41;74	122	102
Size of metastasis	20;21,11	25	30
Treatment of LM	Resection RFA	Laparoscopic lobectomy	Segmental resection

Ca, serum calcium; PTH, parathyroid hormone; PC, parathyroid carcinoma; LM, liver metastases; RFA, radiofrequency ablation.

### Patient 1

A 43-year-old man was diagnosed with pHPT in 2011 due to polydipsia and polyuria, hypercalcemia, severely elevated PTH, and a right parathyroid mass. Primary surgery due to pHPT was performed in May 2011. PC was confirmed histopathologically, and resection margins were tumor free (R0). PTH decreased obviously after the surgery. The patient developed hypocalcemia requiring treatment with intravenous (IV) calcium therapy for 5 days followed by oral calcium supplementation. The patient was subsequently free of disease for 3 years.

In 2014, the patient experienced symptoms similar to those at initial diagnosis of PC with polydipsia, polyuria, and renal stones. Serum calcium was elevated up to 3.74 mmol/L, and PTH was increased to 847 pg/ml. A CT scan suggested a low-density lesion in the left parathyroid gland, multiple small lymph nodes in the neck, and a 15 × 10mm mass in the S5 segment of the right liver. [18F]FDG-PET-CT revealed inflammatory lymph nodes in the neck, but no focal FDG uptake was found in the liver. No significant improvement in hypercalcemia was noted after intramuscular calcitonin injection. The diagnosis of liver metastasis of PC was considered, and then a liver tumor with 2 cm of surrounding liver tissue was resected in October 2014. Calcium decreased immediately from 3.74 mmol/L to 2.64 mmol/L the day after surgery, while PTH dropped to 54.6 pg/ml 3 days later. The histological assessment of the resected tissue confirmed liver metastasis of PC. Histological and immunohistochemical analyses were both consistent with PC (positivity for PTH, loss of AFP). The patient started taking oral calcium tablets and calcitriol daily.

**Figure 1:**
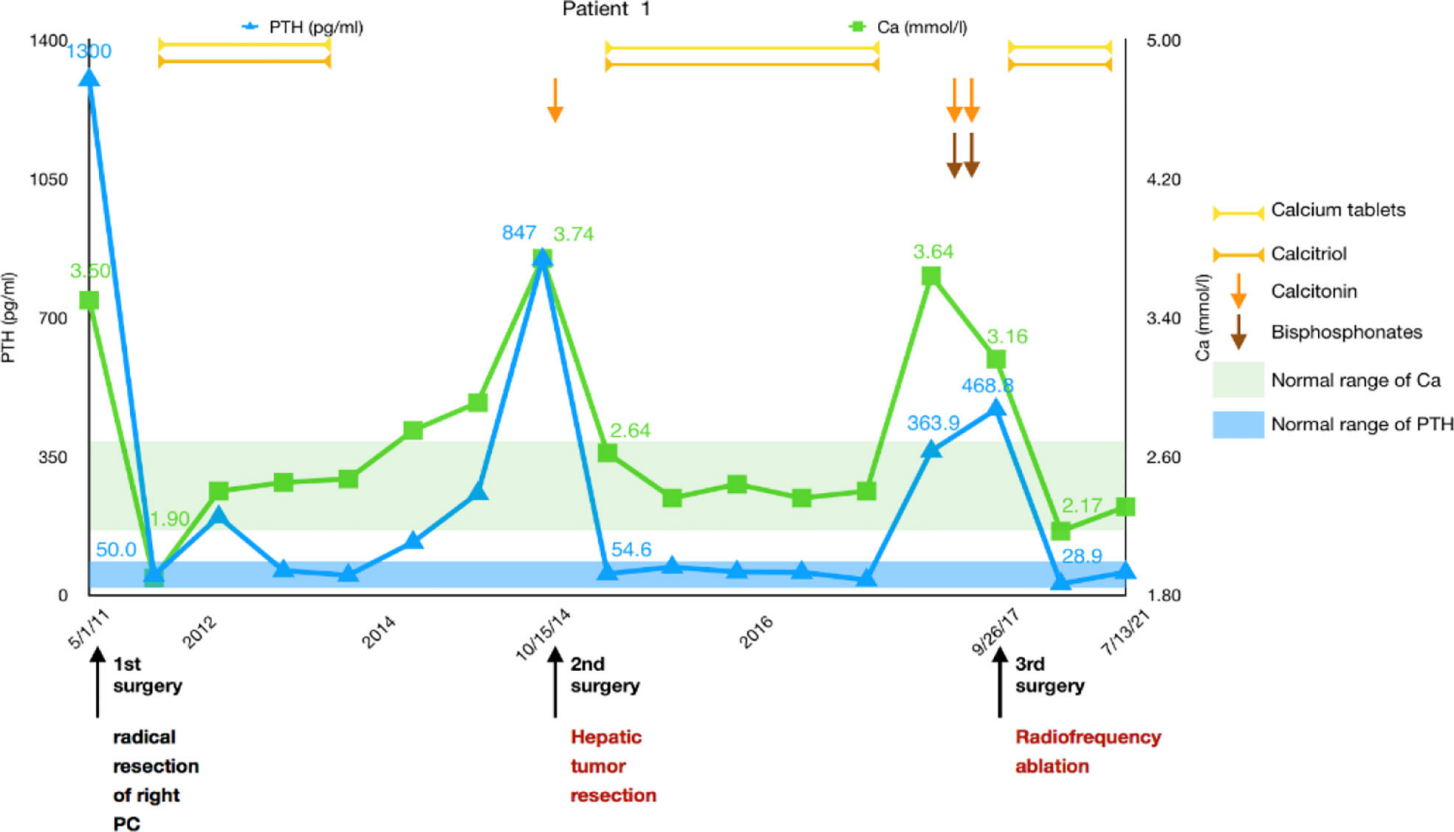
Summary of the clinical course, including changes in serum calcium and PTH of patient 1.

After approximately 3 years, the patient developed symptoms of polyuria, nausea, and rib pain again in August 2017. Serum calcium rose to 3.64 mmol/L, and PTH increased to 363.9 pg/ml. We used bisphosphonates and calcitonin, and the patient’s serum calcium dropped to 3.16 mmol/L, but PTH was still 468.8 pg/ml. No hyperparathyroidism tissue was observed on the enhanced parathyroid CT scan and ^99m^Tc-MIBI scan (MIBI scan), but the abdominal enhanced CT showed two nodules measuring 21 × 13 mm and 11 × 8 mm in the S8 segment of the liver with uneven enhancement, and the possibility of liver metastases was considered. Considering the difficulty of secondary liver surgery and because the patient required minimally invasive treatment, we chose to use radiofrequency ablation (RFA) to treat the two liver lesions in September 2017. On the first day after the operation, serum calcium dropped to 2.17 mmol/L, while PTH was 28.9 pg/ml. We suggested that the patient adhere to the oral administration of calcium tablets, calcitriol, and vitamin D3 and adjust the medications according to serum calcium and PTH levels. No recurrence or metastasis has been found during the follow-up thus far (**Figure 1**).

### Patient 2

A 50-year-old man was diagnosed with pHPT in 2009 due to knee pain, polydipsia, polyuria, hypercalcemia (4.4 mmol/L), severely elevated PTH (more than 2,000 pg/ml), and a right upper parathyroid mass. He underwent a right parathyroidectomy as the initial surgery, which caused PTH to decrease to 180 pg/ml with a serum calcium of 3.0 mmol/L, and his symptoms disappeared for 2 years. The patient experienced similar symptoms again in 2011. Serum calcium and PTH were significantly elevated, and he had undergone a second operation entailing right hemithyroidectomy and right lower and left parathyroidectomy in July 2011. Histological and immunohistochemical analyses showed parathyroid adenoma recurrence. After the operation, the serum calcium level was approximately 3.0 mmol/L, and PTH remained between 500 and 700 pg/ml. Unfortunately, 8 months later, the patient’s serum calcium increased again to 3.72 mmol/L, PTH was 1,164 pg/ml, and he was transferred to our hospital. Ultrasonography revealed several masses measuring from 10 to 23 mm in the right suprasternal fossa and right supraclavicular fossa. The MIBI scan suggested that the masses were hyperfunctional parathyroid tissue; thus, right neck tumor resection was performed in May 2012, and the histology revealed PC with vascular invasion as the first malignant tumor. After 8 months without any symptoms, he experienced polydipsia, polyuria, sickness, and vomiting; his serum calcium level was 3.56 mmol/L; and his PTH level was 1,299 pg/ml. Ultrasonography and MIBI scans showed multiple solid lesions with increased radioactivity at the level of the lower pole of the right thyroid lobe and behind the right common carotid artery measuring 15 to 25 mm. [18F]FDG-PET-CT revealed hypermetabolic foci sized 20 × 25 × 27 mm at the same location, where the average standardized uptake value (SUVavg) was 3.3, and the maximum standardized uptake value (SUVmax) was 5.0. These tumors, including level III and IV lymph nodes, were dissected in March 2013 as the fourth surgery, and pathology confirmed PC recurrence with lymph node involvement. Serum calcium and PTH levels dropped to within the normal range again, and his symptoms disappeared. Until January 2015, the patient’s serum calcium increased to 3.16 mmol/L again, and an ultrasound examination was completed. Two masses were found at the position of the right thyroid lobe and the front of the right internal jugular vein, which measured 17 × 13 × 12 mm and 27 × 21 × 12 mm, respectively. The masses were confirmed to be lymph node metastases of PC after the fifth surgery, and postoperative serum calcium returned to 2.31 mmol/L. Replacement therapy with levothyroxine tablets 50 mg qd was prescribed. One and a half years later, the patient’s re-examination revealed that serum calcium and PTH had increased again, and then an ultrasound examination was arranged. The inner side of the right subclavian vein was found to have a 27 × 21 × 18mm irregular hypoechoic mass, multiple paravertebral artery and paratracheal lesions were observed in the right neck, which ranged from 4 to 9 mm in size, and PC recurrence was considered. These lesions were removed along with the surrounding lymphoid tissues in September 2016, and both intraoperative frozen and postoperative pathology results were considered consistent with the diagnosis of PC recurrence. The patient’s serum calcium decreased to 2.23 mmol/L, PTH became 10.1 pg/ml after the sixth surgery, and no clinical discomfort was reported.

**Figure 2:**
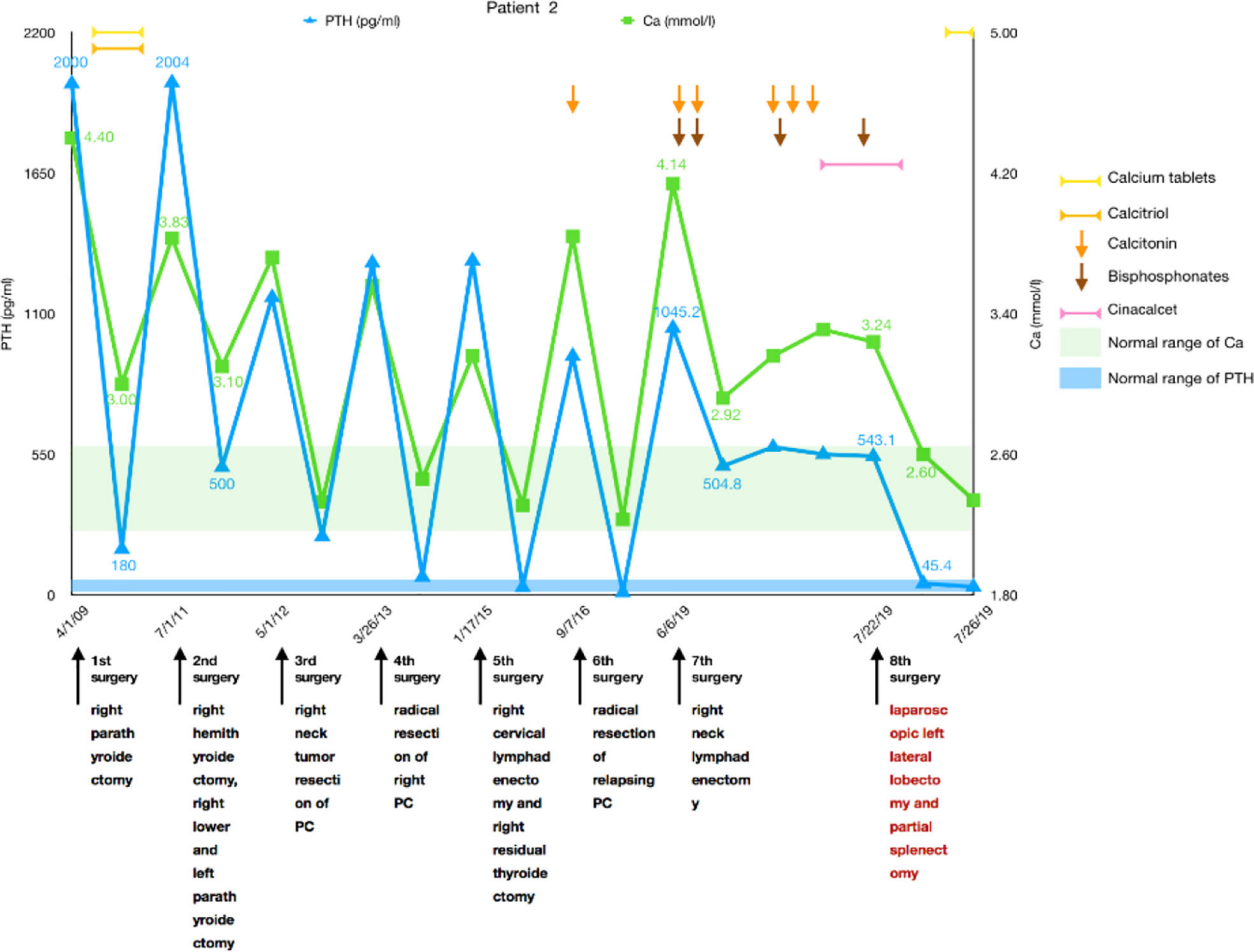
Summary of the clinical course, including changes in serum calcium and PTH of patient 2.

In 2019, the patient had symptoms of anorexia, nausea, vomiting, polydipsia, and polyuria again; his serum calcium was 4.14 mmol/L; his PTH was 1,045.2 pg/ml; and serum calcium improved after calcitonin and bisphosphonate use. Left renal stones were found. Ultrasonography and enhanced parathyroid CT suggested multiple nodular soft tissue-density shadows with mild enhancement in the range of the anterior border of the right sternocleidomastoid muscle, the anterior border of the right internal jugular vein, and between the right common carotid artery and the internal jugular vein at the level of the thoracic inlet. He was considered to have PC recurrence, and right neck lymphadenectomy was performed as the seventh surgery in June 2019. Regrettably, although the patient’s serum calcium decreased to 2.92 mmol/L, PTH did not decrease significantly and remained at approximately 500 pg/ml. No typical improvement was observed despite treatment with physiological saline, diuretics, calcitonin, bisphosphonates, and cinacalcet. MIBI scans and [11C]choline-PET-CT did not reveal another recurrence or distant metastasis; therefore, abdominal enhanced CT and MRI were further performed. Two lesions measuring 25 × 22 mm and 14 × 7 mm were found in the left lateral lobe of the liver and the lower pole of the spleen, respectively, both of which were considered metastatic carcinomas. A laparoscopic left lateral lobectomy and partial splenectomy were immediately performed in July 2019. Histological and immunohistochemical analyses confirmed that the liver lesion was a metastasis of PC. Serum calcium and PTH finally returned to normal ranges after surgery and have been normal during the follow-up to date (**Figure 2**).

### Patient 3

The third patient was a 37-year-old woman with chronic knee pain and hypercalcemia who had undergone three parathyroid-related surgeries and came to our department for metastatic PC management.

The patient had a right mid femur fracture immediately following a squat that required closed reduction and internal fixation in 2013. She was diagnosed with osteoporosis and pHPT due to hypercalcemia (2.8 mmol/L) and elevated PTH (981 pg/ml). She underwent preoperative imaging that revealed a right inferior parathyroid tumor, which had been resected during the initial surgery in August 2013. Histology confirmed an atypical parathyroid adenoma measuring 45 mm. She did not have very regular re-examinations until 5 years later because of polydipsia and polyuria. We found that PTH had increased to 752.8 pg/ml and serum calcium to 2.67 mmol/L. Ultrasound showed multiple solid masses behind and below the right lobe of the thyroid, and an MIBI scan showed uptake in those masses. [18F]FDG-PET-CT showed that the hypermetabolic foci below the isthmus of the thyroid and behind the lower pole of the right lobe were suspicious for malignancy. Radical resection of right PC with right thyroidectomy, isthmus resection, and central nodal clearance was performed in March 2018. Intraoperative PTH dropped from 752.8 pg/ml to 68.5 pg/ml, while serum calcium decreased to 1.92 mmol/L. Histology revealed PC with lymph node metastases. Only 10 months later, the patient was diagnosed with recurrent PC with high levels of serum calcium and PTH, and [18F]FDG-PET-CT showed two hypermetabolic foci behind the left lobe of the thyroid gland measuring 4–18 mm in size. Then, left thyroidectomy and central nodal clearance were performed in her third surgery in February 2019. On the first day after the operation, the serum calcium level decreased from 2.75 mmol/L to 2.46 mmol/L, and the PTH level decreased from 326.9 pg/ml to 8.7 pg/ml. However, histology showed atypical parathyroid adenoma and chronic inflammation of lymph nodes without significant malignancy. Only 2 months later, PTH increased to 184.6 pg/ml, and radiotherapy was carried out in our hospital from April to June 2019 (50 Gy/25 times in the left anterior cervical region and 60 Gy/30 times, 2 Gy/time, 5 times/week in the tumor bed region). Fortunately, PTH dropped to 23.7 pg/ml during the 2 months with normal serum calcium, and then her medication was adjusted to include oral calcium and calcitriol, which was continued for a year until May 2020. Due to elevated blood calcium levels of 2.82 mmol/L, alendronate sodium and vitamin D3 were administered at a weekly cumulative dose of 70 mg to adequately control serum calcium levels. Four months later, PTH increased again, and cinacalcet was required at a dose of 25 mg bid. However, serum calcium levels were not well controlled, and the patient asked to stop taking cinacalcet in December 2020, leaving alendronate sodium and vitamin D3. Six months later, the patient experienced polyuria again, and her serum calcium level increased to 3.16 mmol/L, while her PTH was 896.0 pg/ml. Ultrasonography of the neck and an MIBI scan did not reveal any recurrence, while [11C]choline-PET-CT showed a suspicious hypermetabolic lesion in the superior part of the left thyroid bed just near the carotid artery. Since the lesion was not confirmed to be malignant and was difficult to remove surgically, denosumab was used to replace alendronate sodium and vitamin D3 to control serum calcium levels (2.68 mmol/L).

**Figure 3:**
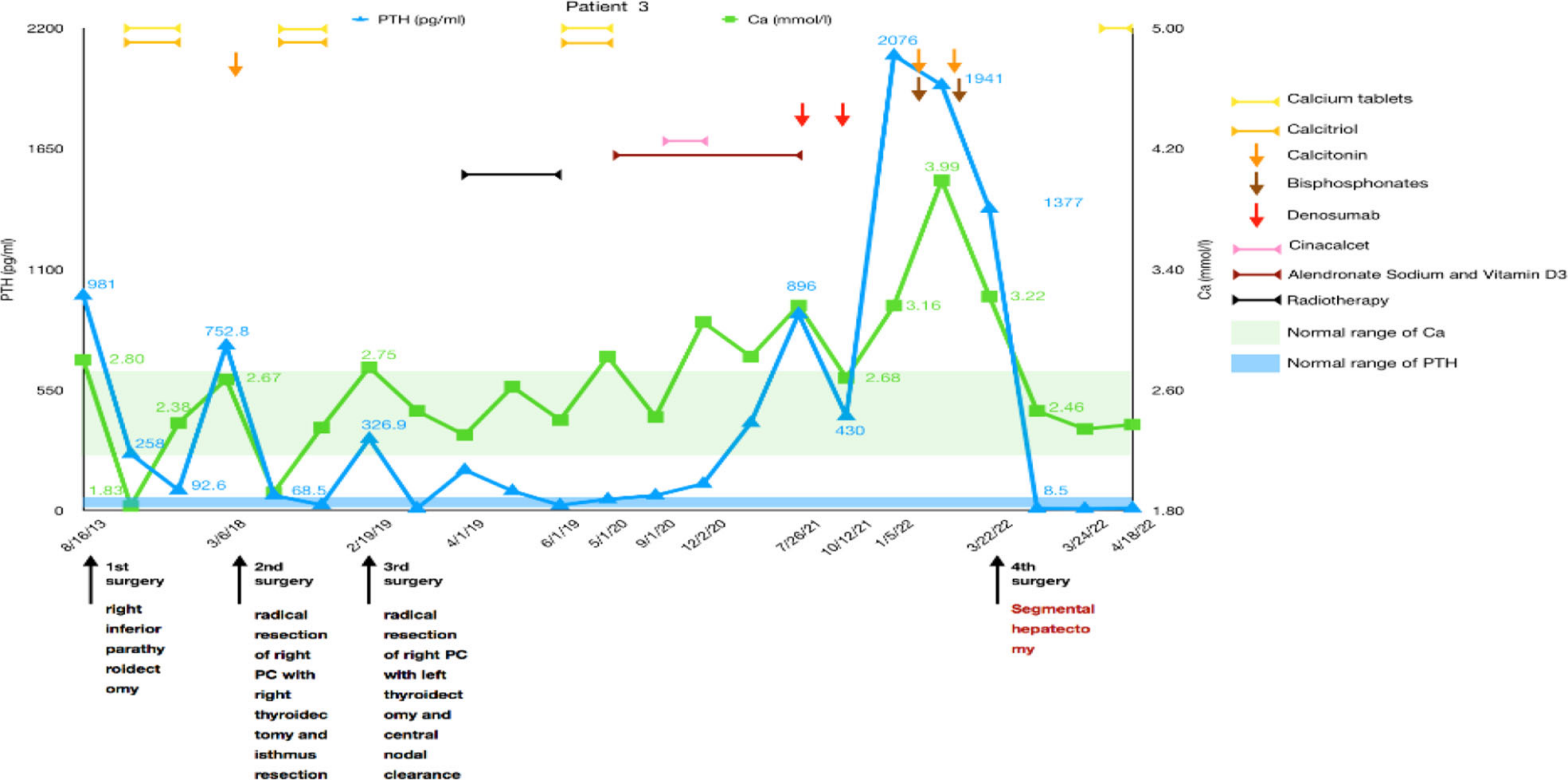
Summary of the clinical course, including changes in serum calcium and PTH of patient 3.

In January 2022, she experienced right knee pain, polydipsia, and polyuria again. Her serum calcium was 3.16 mmol/L, and her PTH was 2,076.0 pg/ml, but no typical hyperfunctional parathyroid tissue was noted on the MIBI scan. Surprisingly, an abnormal mass measuring 30 × 22 mm in size was found in the S6 segment of the right liver by CT and MRI. A diagnosis of liver metastasis of PC was considered, and a segmental hepatectomy was performed in March 2022. Fortunately, her intraoperative serum calcium dropped from 3.99 mmol/L to 2.46 mmol/L, and her PTH decreased from 1941 pg/ml to 8.5 pg/ml. Histological and immunohistochemical analyses were both consistent with PC metastasis. Her serum calcium and PTH levels remained within the normal ranges during the follow-up (**Figure 3**).

### Literature review

**Figure 4:**
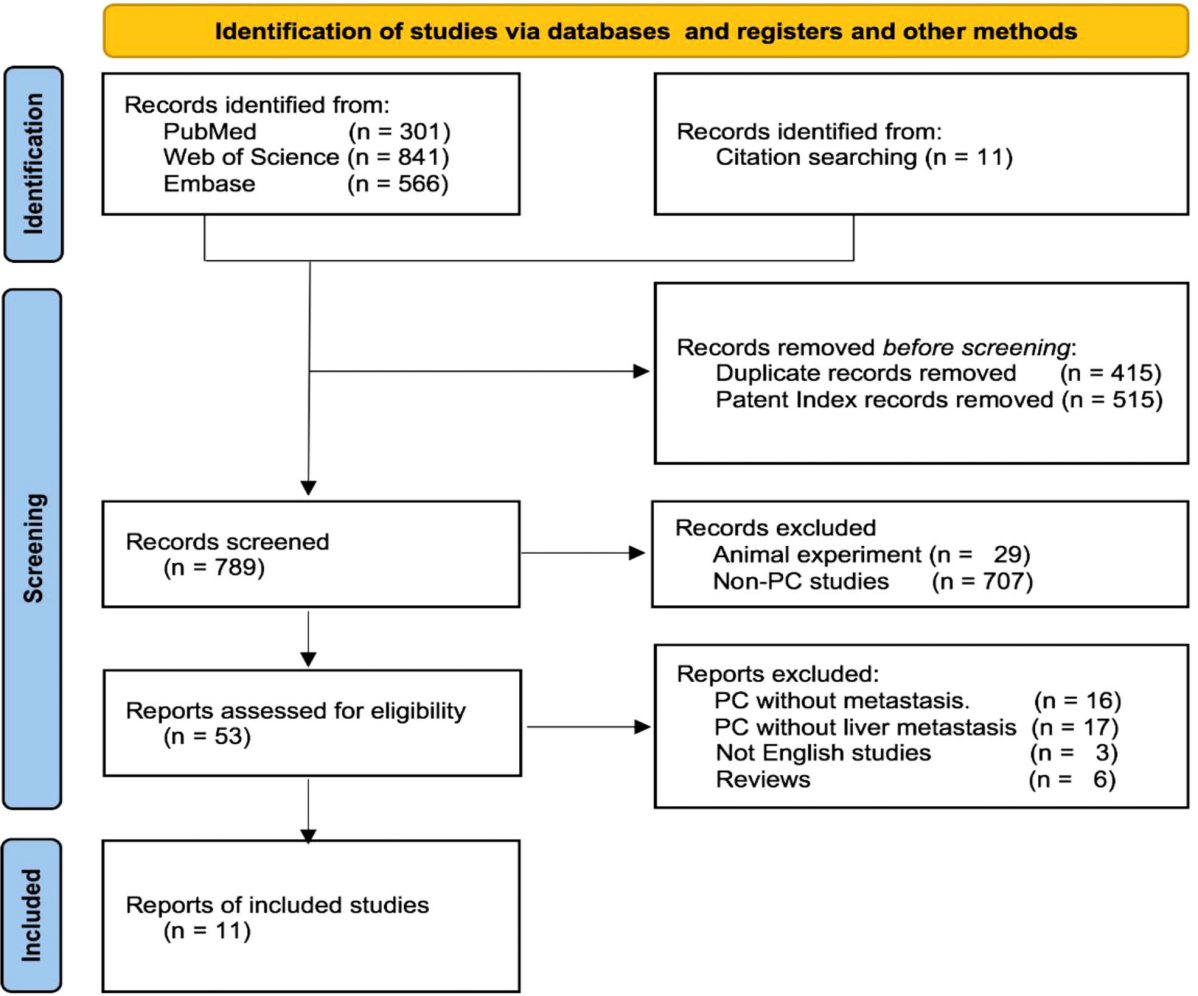
Flow of screening records.

A flow diagram of the study is displayed in **Figure 4** according to the search strategy. Overall, 1,708 articles were initially retrieved, and 11 articles were retrieved from the references. A total of 415 duplicates and 515 patent index records were initially excluded, and then 707 studies without PC and 29 animal experiments were removed by screening the titles or abstracts. Fifty-three studies were identified, and the full texts of these studies were reviewed. Finally, 11 eligible articles were included.

### Patient features

Nine of the 11 included studies were case reports (7–15). A retrospective review analyzed the risk of distant metastasis in 75 PC patients, including 2 patients with liver metastases (4). Another retrospective study published in 1973 reported a total of 70 patients with PC, including 5 with liver metastases, and descriptively analyzed the clinical manifestations, pathology, and prognosis (16).

Sixteen patients in the 11 included studies had liver metastases of PC. An article mentioned the existence of only five patients with liver metastases but lacked relevant data. Therefore, we excluded this article from the data analysis. The detailed information of the last 11 patients and our three cases is summarized in **Table 2**.

**Table 2:** Literature review and our cases of liver metastases of parathyroid carcinoma.

First author	Publication year	Age/Gender	Location/Diameter(mm)	Time to LM (months)	Finding modality of LM	Treatment of LM	Pre→Post Ca (mmol/l)	Pre→Post PTH (pg/mL)	Outcome	Follow-up (months)
Mellagui	2020	54/M	S8/NA	NA	CT/MRI	NA	5.72→NA	2,884→NA	Death	3
Asare	2019			10.8					Death	61
				23.4					Alive	NA
González-Clavijo	2017	74/M	S5, S7/NA	1	PET	TAE	3.01→2.08	1,632→110	Alive	NA
Horie	2010	34/F	Widespread/20	11	CT	Immunization	3.75→NA	120,00→NA	Death	1
Artinyan	2008	71/M	Widespread/NA	6	CT/biopsy	RFA/TAE	3.18→2.18	266→53	Alive	4
Mezhir	2007	72/F	S6/20	96	CT/biopsy	Segmental resection	4.5→2.5	597→67	Alive	2
Szmuilowicz	2006	54/M	Widespread/NA	NA	CT/biopsy	Cinacalcet	4.2→2.0	327→263	Death	2
Ahmad	2005	42/F	S4a/45	NA	MRI/MIBI/biopsy	Laparoscopic microwave ablation	4.01→2.72	955*→46.4	Alive	15
van Haaren	1996	35/F	S7-8/30	36	CT	Segmental resection	5.0→2.5	609→NA	Death	60
Krudy	1982	48/M	Right lobe/20	36	CT	Resection	3.7→NA	5,000→NA	Alive	NA
Patient 1		43/M	S5/20 S8/21,11	41 74	CT/MRI	Resection RFA	3.74→2.64 3.64→2.17	847→54.6 468→28.9	Alive	92
Patient 2		50/M	S2/25	122	CT/MRI	Laparoscopic lobectomy	3.24→2.60	543→45.4	Alive	35
Patient 3		37/F	S6/30	102	CT/MRI	Segmental resection	3.99→2.46	1,941→8.5	Alive	3

M, male; F, female; LM, liver metastases; Ca, serum calcium; PTH, parathyroid hormone; Pre, before treatment; Post, after treatment; NA, not available; PET, [18F]FDG-PET-CT; TAE, transcatheter arterial embolization; RFA, radiofrequency ablation; MIBI, ^99m^Tc-MIBI scan.

### The incidence of liver metastases in PC patients

Asare (4) reported that the incidence of liver metastases was 2/75 (2.67%), and Schantz (16) showed that the rate was 5/70 (7.14%), while the incidence at our center was 3/254 (1.18%). Three patients had liver metastases at the time of PC diagnosis, and the other 11 suffered liver metastases at 1–122 months after initial identification, with a median of 36 months.

### Diagnosis of liver metastases in PC patients

**Figure 5:**
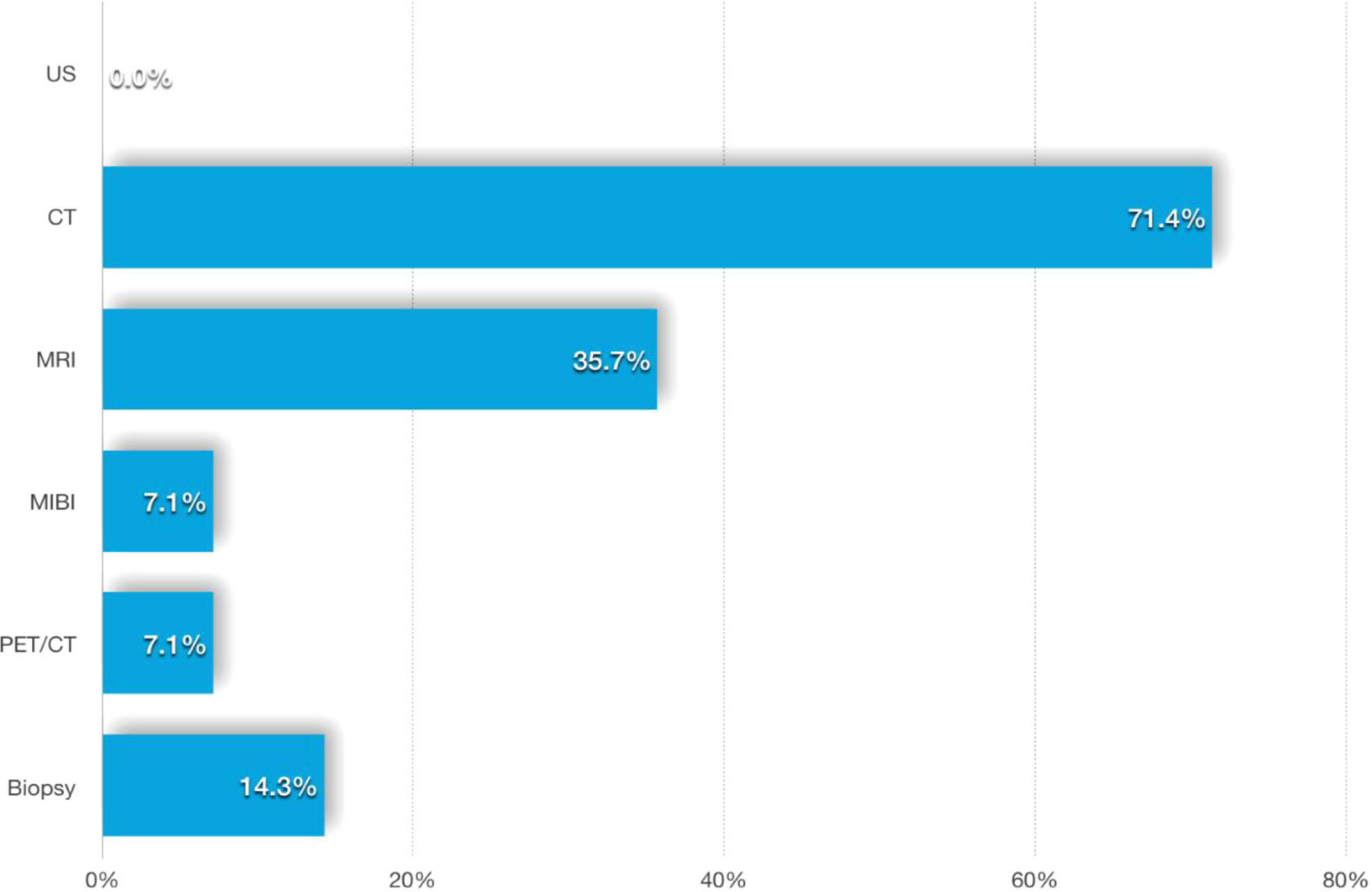
Different methods for locate the liver metastases.

**Figure 6:**
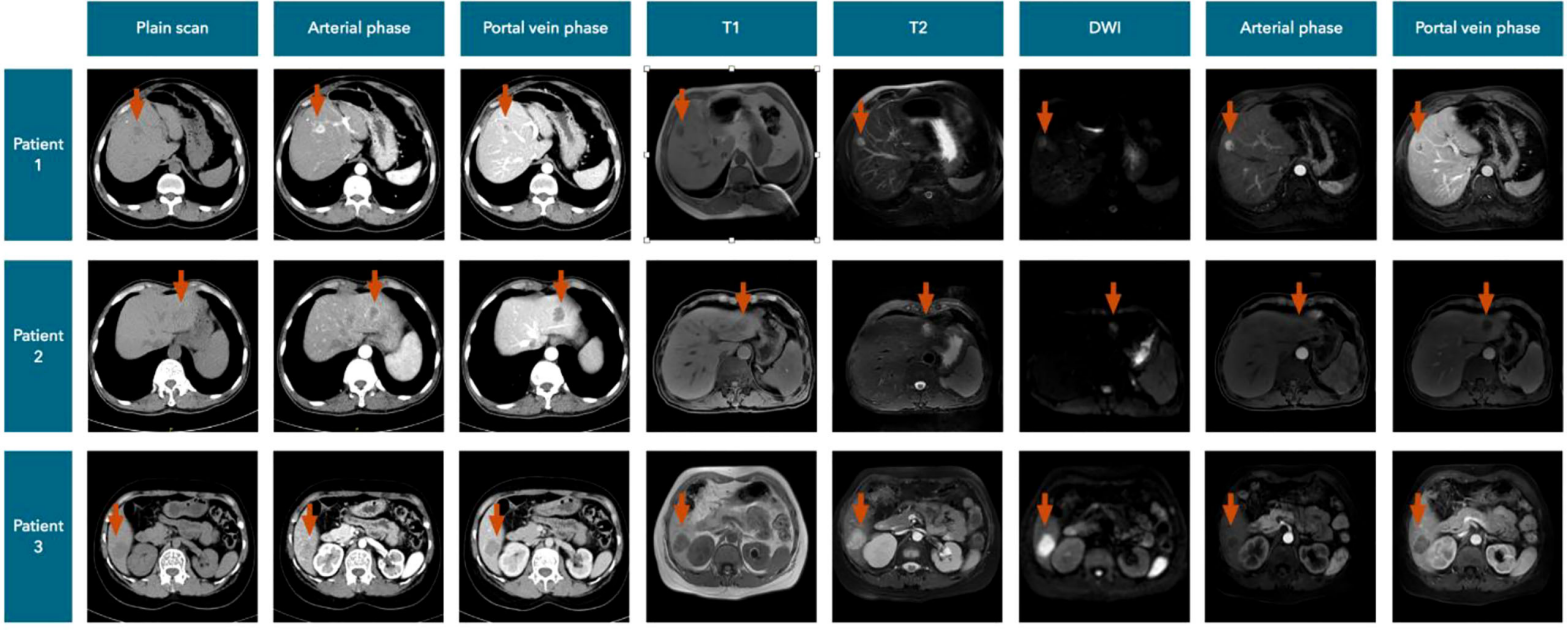
CT and MRI images of three patients.

Among the 14 liver metastatic patients, none mentioned liver lesion detection by abdominal ultrasonography. Most of the patients’ liver lesions were found by abdominal CT scans, and approximately one-third of the lesions could also be detected by MRI. MIBI and [18F]FDG-PET-CT imaging appear to be less sensitive to liver metastases, while two of the four patients with FNAB had a positive result (**Figure 5**). The CT and MRI images of our three patients are listed in **Figure 6** for comparison.

Liver metastases are hematogenous and more prone to multifocal metastases. Three of the 14 patients had widespread metastases at the time of PC diagnosis, 2 did not have lesion site data, and 3 had tumors in different or across segments. The diameter of the metastases ranged from 11 mm to 45 mm, with an average of 24.2 mm.

### Treatment and follow-up of liver metastases in PC patients

Patients with multiple metastatic lesions often miss the opportunity for surgical treatment. Among the three patients with widespread metastases, only one received RFA combined with transcatheter arterial embolization (TAE) treatment, and two received immunotherapy and cinacalcet. Among the three patients with multisegmental metastases, two underwent surgical resection, and one patient underwent TAE. In total, six patients had isolated liver metastasis; five patients underwent surgical resection, RFA, or microwave ablation; and one patient was prescribed RFA, but he refused.

Of the 14 patients, 5 patients died during the follow-up, and the overall survival rate was 64.28%. Metastases were extensive when identified in two patients, who refused RFA 3 months later. One patient did not have treatment method data, and one patient died after surgical resection. Of all nine patients who underwent surgery, RFA or TAE, only one patient died after a follow-up of 60 months.

## Discussion

Because of the rarity of PC and the absence of typical symptoms and signs, the diagnosis and treatment of PC involve serious challenges (1). The most common manifestations are severe hypercalcemia and inappropriately elevated PTH. Based on the three patients and the literature, the possibility of malignant pHPT should be considered when the following clinical features exist: (a) markedly elevated calcium levels typically above 3 mmol/L (17); (b) PTH levels above 3 to 10 times the upper limit of normal (3, 18, 19); (c) a palpable neck mass with a lesion >30 mm (20); (d) severe symptoms of bone (osteitis fibrosa cystica) disease and renal disease (renal stones and nephrocalcinosis) (3, 18); and (e) large, inhomogeneous, hypoechoic, and lobulated masses on ultrasonography. In recent guidelines for parathyroid imaging, the European Association of Nuclear Medicine (EANM) pointed out that ^99m^Tc-MIBI is the first-line imaging tracer in pHPT, suggesting that ^99m^Tc-MIBI SPECT/CT combined with cervical ultrasound (cUS) can achieve a sensitivity up to 95% for functional parathyroid glands. [18F]Fluorocholine-PET-CT is considered to be an alternative first-line imaging method, particularly in cases of negative or inconclusive conventional imaging methods. As en bloc resection of PC during the initial operation directly affects the prognosis (21, 22), surgeons must be extra careful in patients with these similar conditions.

PC recurrence and metastasis are difficult to avoid, and 33%–82% of patients will reportedly relapse (17, 23, 24), similar to our three patients who all suffered multiple recurrences (two to seven times). Approximately 25%–30% of patients develop distant metastases at some point in the course of the disease. The most common sites of distant metastases are the lungs (40%) and liver (10%), but they also spread to the abdominal lymph nodes, bones, pleura, pericardium, and pancreas in rare cases (3, 4). Apart from the lungs, the liver is the second most common target organ for distant metastases. Qiu summarized 38 patients with distant metastasis in 27 studies, with liver metastases identified in 13.16% (25). We believe that these differences are related to an insufficient sample size. Nonetheless, the diagnosis and treatment of this group of patients are rarely mentioned. Based on the patients at our center and a literature review, we tried to summarize some characteristics that may be useful for both diagnosis and treatment.

### Diagnosis of liver metastases

Most local recurrences and distant metastases have been reported to occur 2–3 years after the initial surgery, but some cases of recurrence develop after 23 years (3). Based on our data, liver metastases appeared 1–122 months after the initial PC diagnosis, with a median of 36 months. The time to liver metastasis development after PC surgery is not very certain; therefore, we suggest that postoperative reviews should be carried out routinely.

Most liver metastases of PC are functional, and even a single liver metastasis will have some clinical manifestations of hypercalcemia, which decreases quality of life and survival (4). However, most of the patients lack specific abdominal symptoms, and more reliance on laboratory tests and imaging studies is necessary to diagnose liver metastases.

Ultrasound is always valuable as an economical and noninvasive means of examination, is significant for the diagnosis of neck lesions, and has better sensitivity upon combination with ^99m^Tc-MIBI SPECT/CT, but it is not sensitive for the detection of liver metastases (20, 26). In 7 of 14 patients, neck lesions were found by ultrasound, but no liver masses were detected by ultrasound. However, ultrasound-guided biopsy of liver lesions played a role, and two of four cases were clearly diagnosed as metastases (10–12). Ultrasound guidance was used in three patients who received RFA or microwave ablation, and liver lesions that were difficult to find during surgery could also be located by intraoperative ultrasound scanning (11, 13).

A total of 71.4% of the 14 patients had liver lesions detected by contrast-enhanced CT and/or MRI, and we therefore considered these modalities to be more useful for discovering liver metastases, but imaging manifestations were rarely mentioned in the published literature.

According to our cases, we summarized the CT manifestations of liver metastases of PC (**Figure 6**). Plain CT scans showed (a) hypodense focal lesions with hazy borders, (b) uneven enhancement with peripheral rim enhancement in the arterial phase, and (c) obviously decreased enhancement with lower density compared to that in the normal liver in the portal vein and delayed phases.

MRI is another important imaging test, and the metastases always showed (a) long T1 and slightly longer T2 signals, (b) hyperintensity or high ring-shaped signal intensity on diffusion-weighted imaging (DWI), (c) inhomogeneous enhancement in the arterial phase and halo ring-like enhancement, and (d) enhanced attenuation in the portal vein and delayed phases.

PET/CT is commonly used to detect recurrence or metastasis of malignancies, but its role in PC has not been clearly reported, and only a few case series studies show the benefit of PET scans, especially in the postoperative period, to detect local recurrence and distant metastases, such as subcutaneous recurrence and breast metastasis (27–29). Only one patient’s liver lesion was found by [18F]FDG-PET-CT among all the cases (8); such results challenge the sensitivity of [18F]FDG in the diagnosis of liver metastases. [18F]Fluorocholine ([18F]FCH)-PET-CT (or PET-MR) is a new method that can accurately detect parathyroid adenoma, is considered a “one-stop-shop” method for preoperative parathyroid localization in patients with pHPT, and may have good application prospects for the detection of parathyroid cancer and metastasis (26, 30, 31).

In recent years, [11C]choline-PET/CT has been increasingly used to localize parathyroid lesions causing pHPT, especially when diagnosing parathyroid adenoma (32–34). We tried this examination but failed to identify liver lesions. Thus, further research is necessary.

When the nature of metastatic lesions cannot be determined by various imaging examinations, fine-needle aspiration biopsy (FNAB) of metastatic liver lesions may be considered appropriate, although potential needle tract seeding challenges the role of FNAB in neck lesions (21, 35).

### Treatment of liver metastases

Patients with PC who have developed liver metastases often have polyuria, polydipsia, and hypercalcemia. Although resection of liver metastases is palliative surgery, it often improves hypercalcemia and symptoms. The main goal of systemic therapy is to temporarily control symptoms and improve hypercalcemia crisis to reduce and stabilize serum calcium and thus create conditions for surgery (3). Physiological saline, diuretics, calcitonin, and bisphosphonates are the most commonly used drugs, which have been clinically demonstrated to lower serum calcium but often only temporarily. The calcimimetic drug cinacalcet is another option that combines with calcium-sensing receptors to reduce PTH secretion and control serum calcium (29). It can be considered for hypercalcemic patients with inoperable PC, incomplete resection of PC lesions, or postoperative recurrence and effectively lowers serum calcium levels in approximately two-thirds of patients (36, 37), but unfortunately, our liver metastatic patients have not shown satisfactory outcomes. Additionally, some case reports considered that monthly denosumab injection can maintain serum calcium at lower levels, although the effect is transient (38, 39).

As mentioned in some studies, severe hypercalcemia is an important risk factor affecting mortality, while most metastatic lesions are functional. Surgery entails the removal of all functioning tumor tissue to lower serum calcium and improve symptoms, although it has not unanimously been shown to lead to survival benefits (25, 29, 40–42).

The number of liver metastatic lesions in our three patients was small, and the sites were relatively limited to one segment. These characteristics created a good condition for surgical resection. During the operations, we removed liver tissue, including the tumor and more than 2 cm around the tumor. If conditions permitted (two of three patients), extralateral tissue including these areas was completely resected, or a segmental hepatectomy was performed. The serum calcium and PTH levels of these three patients were all significantly decreased once the tumor had been removed, even intraoperatively or the day after surgery, and systemic symptoms were also significantly improved.

Based on our experience and some literature (11, 14), we suggest that the surgical scope of PC liver metastases should include the liver tissue more than 2 cm around the tumor as much as possible, and segmental hepatectomy is also possible regardless of whether the surgery is performed *via* laparoscopy or laparotomy. Similar to the initial surgery for PC, the integrity of the tumor capsule must be preserved during the operation to avoid implantation metastasis.

Unfortunately, not all patients with liver metastases have the opportunity to undergo surgery, such as those with multiple metastatic tumors occurring in different liver segments (10). Multiple abdominal operations lead to surgical difficulties (patient 1), and such patients could not endure or were not willing to undergo surgery due to their physical conditions (7). At this time, less invasive RFA may be more suitable than surgical resection. Our patient 1 had undergone CT-guided percutaneous RFA of the tumors and remains in biochemical remission for 54 months thus far. The case reported by Artinyan (10) was treated with combined RFA and TAE because of widespread liver metastases, and the patient was followed up for 4 months and showed normal PTH and serum calcium levels. Ahmad reported another case of S4a liver metastasis treated by laparoscopic microwave tissue ablation and achieved at least 15 months of survival (13). A few published studies have pointed out the possible benefits of RFA in the treatment of distant metastases, and some patients survive for up to 17 years with a combination of several RFA medical treatments (10, 43, 44). Although no overwhelming evidence indicates that RFA prolongs survival, it destroys tumor cells and effectively controls serum calcium and PTH levels, thereby improving symptoms; therefore, we consider it to be a useful treatment for liver metastases.

**Figure 7:**
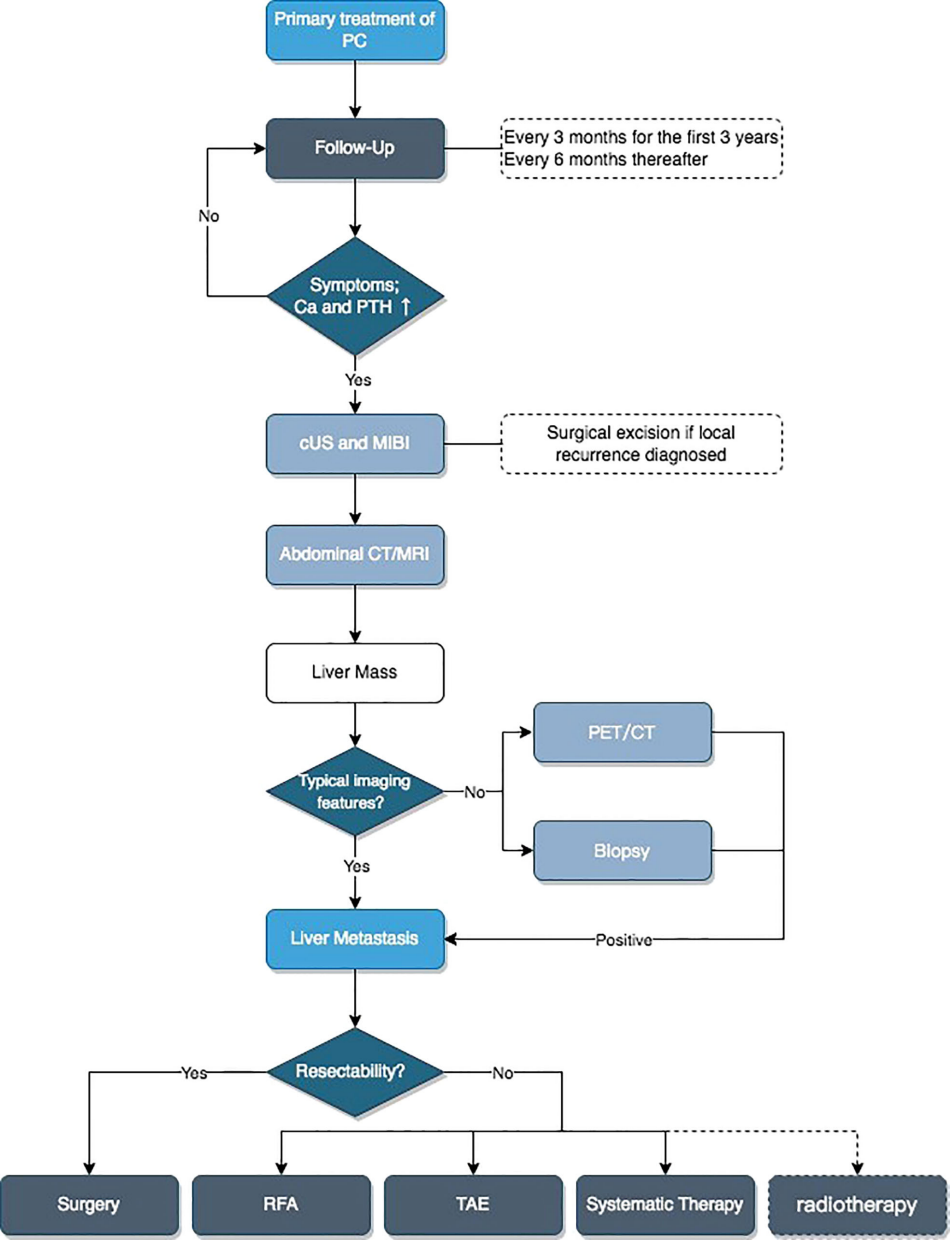
Flowchart for diagnosis and treatment of liver metastatic patients. Ca, serum calcium; PTH, parathyroid hormone; cUS, cervical ultrasound; MIBI, ^99m^Tc-MIBI scan; CT/MRI, contrast-enhanced CT and/or MRI; PET/CT, [18F]FDG-PET-CT or [18F]FCH-PET-CT or [11C]choline-PET-CT; RFA, radiofrequency ablation; TAE, transcatheter arterial embolization; Systematic Therapy, including physiological saline, diuretics, calcitonin, bisphosphonates, cinacalcet, or denosumab.

Based on the above content, we developed a flowchart for the diagnosis and treatment of patients with liver metastases of PC (**Figure 7**).

## Conclusions

PC is a rare endocrine malignant tumor that is very difficult to diagnose preoperatively and is prone to recurrence or distant metastasis. The liver is the second most common target organ for distant metastases of PC, and enhanced CT and MRI can provide a valuable diagnostic basis. Whether [18F]FDG-PET-CT, [18F]FCH-PET-CT, or [11C]choline-PET-CT can be used as a diagnostic basis for liver metastases requires further study. Liver metastases can be removed by laparotomy or laparoscopy, and we suggest that the scope of resection should include the liver tissue more than 2 cm around the tumor as much as possible, and segmental hepatectomy is also possible. In addition, RFA is also a method worth considering in the management of distant metastases. Of course, for such rare endocrine tumors, interdisciplinary prospective controlled studies are still needed to demonstrate the effectiveness of these measures.

## Data availability statement

The raw data supporting the conclusions of this article will be made available by the authors, without undue reservation.

## Ethics statement

Ethical review and approval was not required for the study on human participants in accordance with the local legislation and institutional requirements. The patients/participants provided their written informed consent to participate in this study.

## Author contributions

CS and JZ conceived the manuscript. All authors reviewed the manuscript. All authors contributed to the article and approved the submitted version.

## Funding

This study was funded by CAMS Innovation Fund for Medical Sciences (CIFMS) (No. 2020-I2M-CT-B-026).

## Acknowledgments

We thank native English-speaking editors at AJE for correcting the language in this study.

## Conflict of interest

The authors declare that the research was conducted in the absence of any commercial or financial relationships that could be construed as a potential conflict of interest.

## Publisher’s note

All claims expressed in this article are solely those of the authors and do not necessarily represent those of their affiliated organizations, or those of the publisher, the editors and the reviewers. Any product that may be evaluated in this article, or claim that may be made by its manufacturer, is not guaranteed or endorsed by the publisher.
